# Supplementary material for: Evaluation of Endobronchial Ultrasound-Guided Transbronchial Needle Aspiration (EBUS-TBNA) Samples from Advanced Non-Small Cell Lung Cancer for Whole Genome, Whole Exome and Comprehensive Panel Sequencing
Source: Cancers (Basel). 2024 Feb 15;16(4):785. doi: 10.3390/cancers16040785 (PMC10887389; doi:10.3390/cancers16040785)
Supplement: Supplementary file 1 [file cancers-16-00785-s001.zip › Supplementary Figures.pdf]

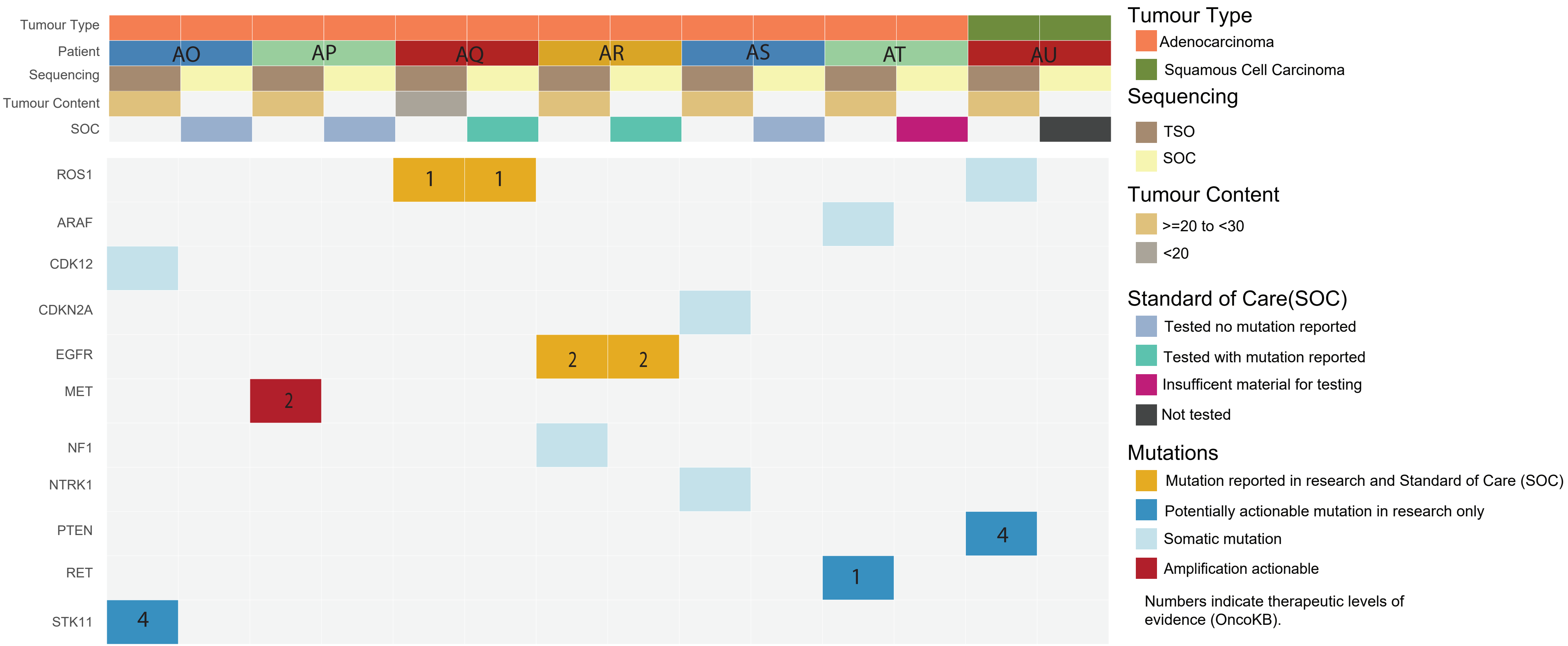

Figure S1: Oncoplot of mutations detected in seven cases with <30% tumour content using TSO500 panel sequencing compared to the mutations reported by diagnostic testing on matched FFPE material obtained from the same EBUS-TBNA procedure. Patients indicated by letters and each column reports results for TSO500 and SOC. Patients are ordered by tumour type. Actionable genes were selected from evidence reported on OncoKB MSK's Precision Oncology Knowledge Base (consulted on 09/08/23). Genes are included if a mutation was detected in at least one tumour. Numbers reported in the plot indicate therapeutic levels of evidence for the mutation identified (1 – Tier 1: FDA recognized and approved drug, 2 - Tier 2: standard of care by professional guidelines to an approved drug, 3 – Tier 3: Investigational, with clinical evidence, 4 – Tier 4: Hypothetical, biological evidence).

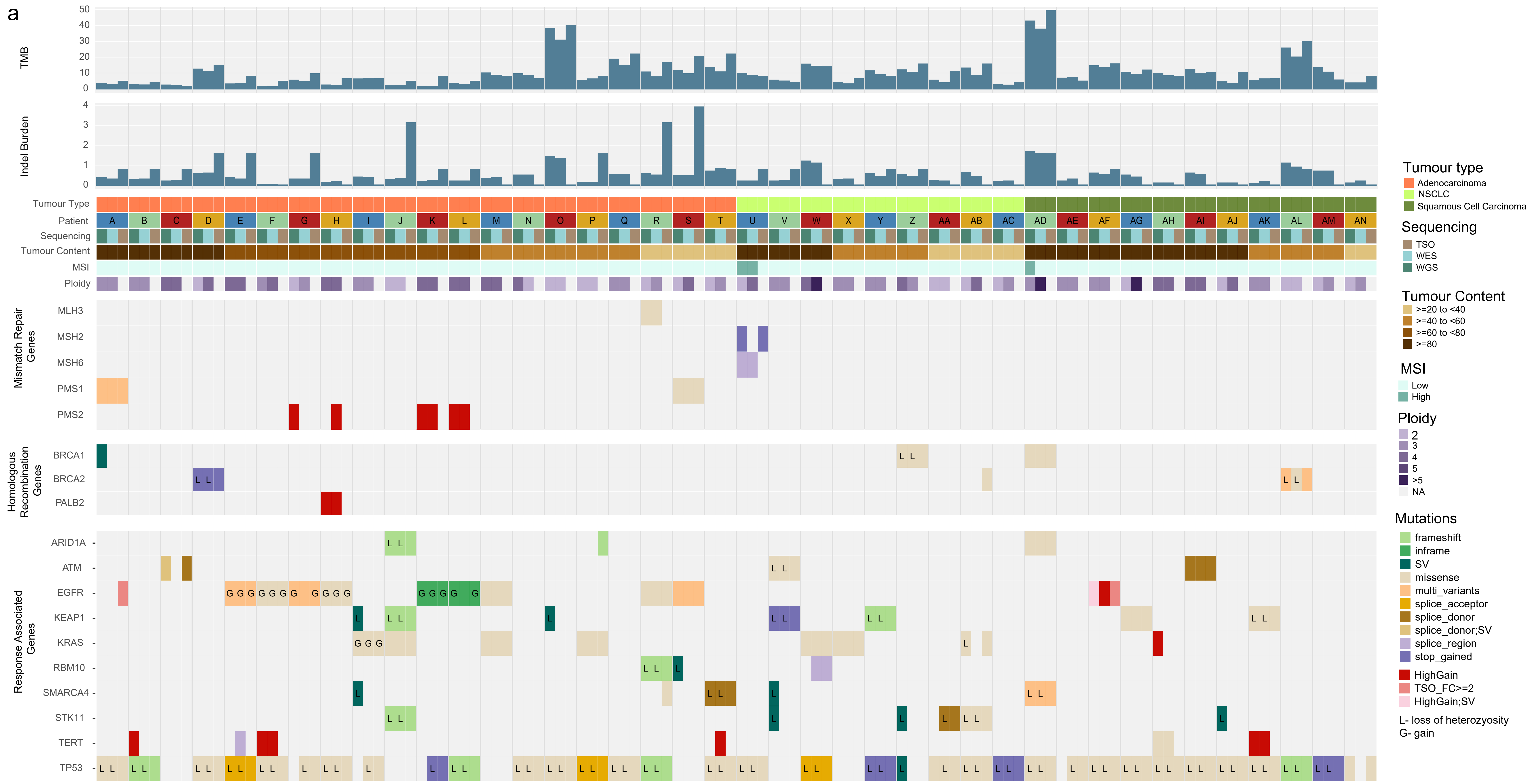

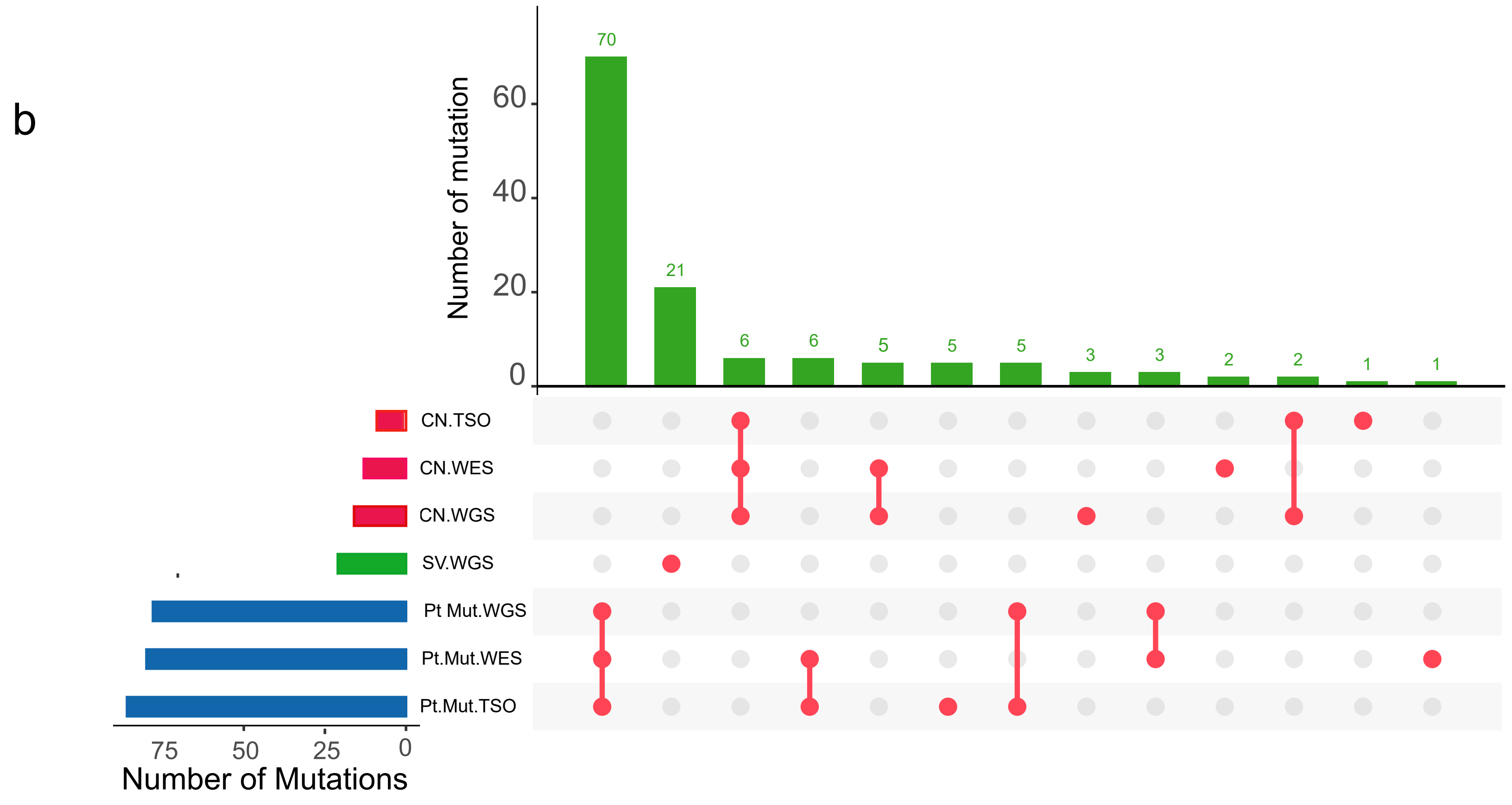

Figure S2: Putative genomic biomarkers of response to immunotherapy. The same DNA extraction was sequenced by WGS, WES and TSO500. A) Patients are represented by letters, with three sequencing platforms. Top two plots show tumour mutation burden (TMB) and the indel burden estimated using mutations detected by each sequencing platform per case. Lower three plots show mutations n mismatch repair genes, homologous recombination genes that have been associated with high mutation burden i n tumours followed by genes previously reported as potential biomarkers for response to immunotherapy treatment. Mutation types are described in the legend. B) Upset plot with number of mutations called by all three sequencing platforms, or by two sequencing platforms or unique to each sequencing platform.

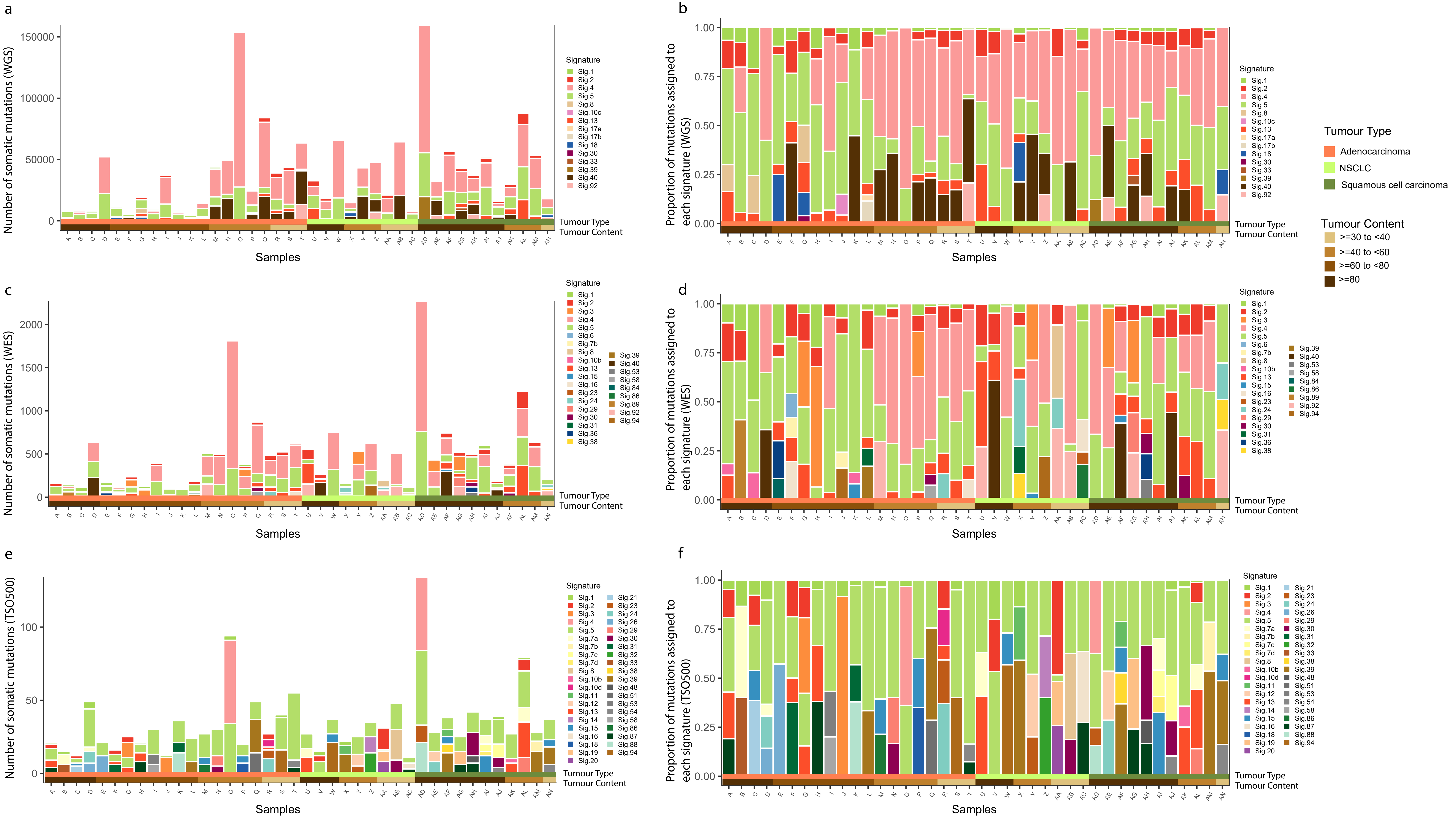

Figure S3: Mutational signatures extracted from mutations detected in each tumour sample by each sequencing platform (WGS, WES or TSO500). Plots on the left show the number of mutations assigned to mutational signatures in each sample; while plots on the right show the proportion of somatic mutations assigned to each signature. Plots A and B show signatures extracted from WGS data; plots C and D show signatures extracted from WES data; plots E and F show signatures extracted from TSO500 data. Each plot is ordered by tumour subtype Adenocarcinoma (Orange), NSCLC (light green) and Squamous Cell Carcinoma (dark green), then within each tumour type samples were ordered by tumour content estimated by SNP arrays (high to low) represented at the bottom of each plot.

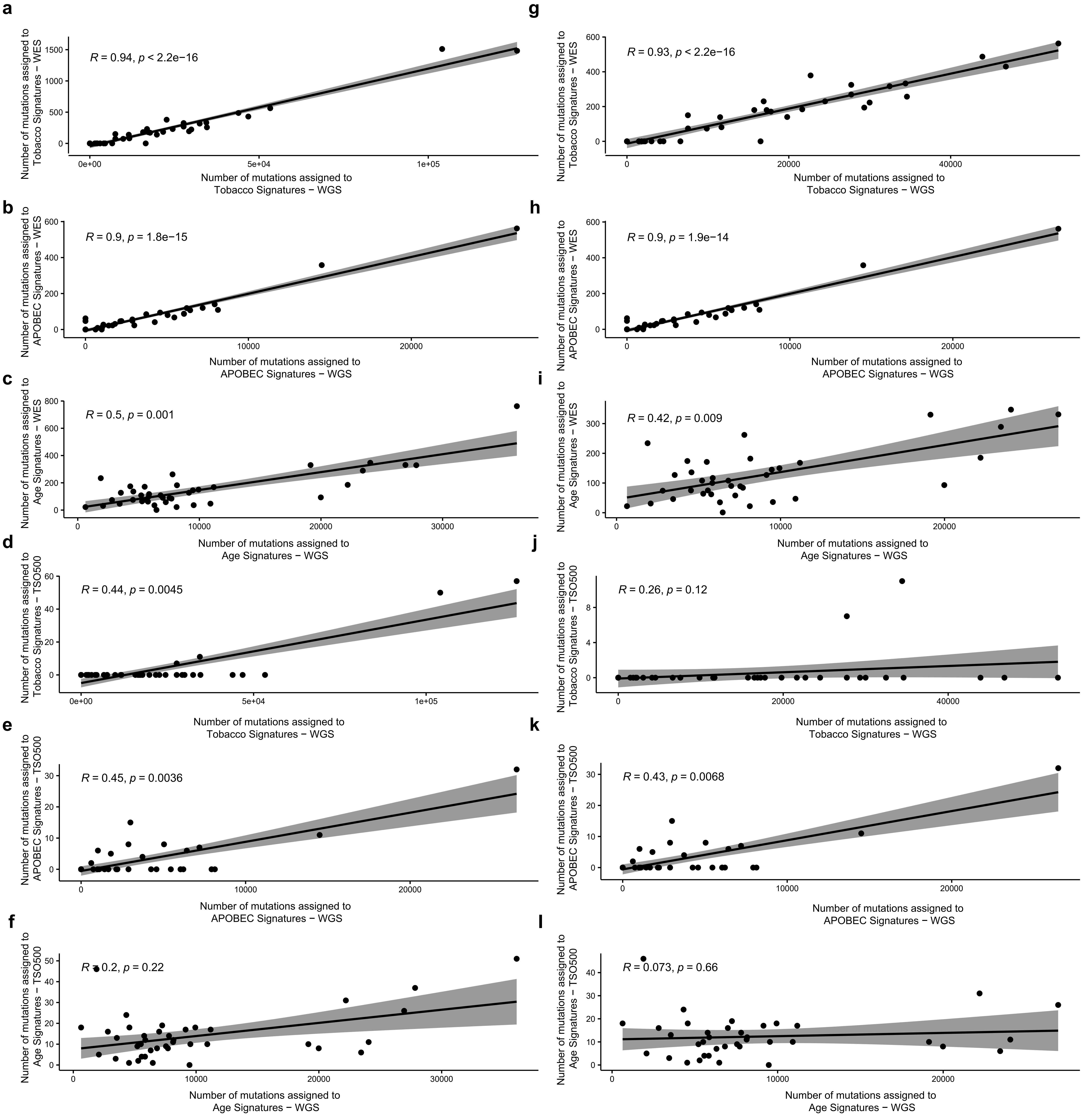

Figure S4: Cross sequencing platforms correlations of the number of mutations assigned to tobacco, APOBEC and clock-like/age mutational signatures. Spearman correlation across the 40 samples cohort between number of mutations assigned to A) tobacco signatures in WGS and WES data; B) APOBEC signatures in WGS and WES data; C) clock-like/age signatures in WGS and WES data; D) tobacco signatures in WGS and TSO500 data; E) APOBEC signatures in WGS and TSO500 data; F) clock-like/age signatures in WGS and TSO500 data. G to L) Spearman correlations of mutations assigned to tobacco, APOBEC and age signatures between sequencing platforms as through A to F but after removing two samples with a high mutation load (>30 mutations/Mb).
